# Indigenous Perspectives and Gene Editing in Aotearoa New Zealand

**DOI:** 10.3389/fbioe.2019.00070

**Published:** 2019-04-11

**Authors:** Maui Hudson, Aroha Te Pareake Mead, David Chagné, Nick Roskruge, Sandy Morrison, Phillip L. Wilcox, Andrew C. Allan

**Affiliations:** ^1^Faculty of Māori and Indigenous Studies, University of Waikato, Hamilton, New Zealand; ^2^Independent Researcher, Wellington, New Zealand; ^3^Plant and Food Research, Palmerston North, New Zealand; ^4^School of Agriculture and Environment, Massey University, Palmerston North, New Zealand; ^5^Faculty of Māori and Indigenous Studies, University of Waikato, Hamilton, New Zealand; ^6^Department of Mathematics and Statistics, University of Otago, Dunedin, New Zealand; ^7^Plant and Food Research, Auckland, New Zealand; ^8^School of Biological Sciences, University of Auckland, Auckland, New Zealand

**Keywords:** indigenous, gene editing, Māori, ethics, regulation, New Zealand

## Abstract

Gene editing is arguably the most significant recent addition to the modern biotechnology toolbox, bringing both profoundly challenging and enabling opportunities. From a technical point of view the specificity and relative simplicity of these new tools has broadened the potential applications. However, from an ethical point of view it has re-ignited the debates generated by earlier forms of genetic modification. In New Zealand gene editing is currently considered genetic modification and is subject to approval processes under the Environmental Protection Authority (EPA). This process requires decision makers to take into account Māori perspectives. This article outlines previously articulated Māori perspectives on genetic modification and considers the continuing influence of those cultural and ethical arguments within the new context of gene editing. It also explores the range of ways cultural values might be used to analyse the risks and benefits of gene editing in the Aotearoa New Zealand context. Methods used to obtain these perspectives consisted of (a) review of relevant literature regarding lessons learned from the responses of Maori to genetic modification, (b) interviews of selected ‘key Maori informants’ and (c) surveys of self-selected individuals from groups with interests in either genetics or environmental management. The outcomes of this pilot study identified that while Māori informants were not categorically opposed to new and emerging gene editing technologies *a priori*, they suggest a dynamic approach to regulation is required where specific uses or types of uses are approved on a case by case basis. This study demonstrates how the cultural cues that Māori referenced in the genetic modification debate continue to be relevant in the context of gene editing but that further work is required to characterize the strength of various positions across the broader community.

## Background

### Gene Editing

All living organisms contain long molecules of DNA which are inherited between generations. The total sum of DNA from an organism is referred to as its “genome,” which itself includes all of its “genes.” An organism's DNA affects how it looks and how it behaves. DNA can change spontaneously, generating new “mutations” (or “variants”) that can have a visible effect. For example several mutations of the eye color character have occurred during human history. Similarly, key mutations in traits such as grain yield and milk production selected by farmers have contributed to crop domestication and agriculture. In the last century and since the discovery of the structure and importance of DNA as the molecule encoding life, several techniques have been developed to artificially alter genes and genomes. The latest of such technique is “gene editing.”

Gene editing is a technology that enables scientists to alter the DNA of an organism in a very precise way. The technique relies on the CRISPR-Cas9 (clustered, regularly interspaced, short palindromic repeats—associated protein Cas9) system that is capable of recognizing a specific DNA motif in the genome. The Cas9 protein then cuts it the DNA sequence to produce double stranded breaks, which can be fixed by the repair system in a non-homologous end joining manner with variable sizes of insertions or deletions and therefore generates DNA mutations (Jinek et al., 2012; Chang et al., 2016). Gene editing has some key differences with other techniques used to generate DNA mutations. Radiation-based mutation using gamma-ray irradiation generates many DNA mutations across the genome in a random fashion. Radiation is used extensively in plant breeding to generate new traits such as seedless table grapes. Unlike radiation, gene editing only targets a precise location in the genome. Another method that has been used in the last 40 years is genetic modification using transgenics (often referred to as GM or GMOs). The principle of transgenics is to insert an entire gene into the host genome. The inserted gene often comes from a different organism. For example, insect-resistance Bt maize, eggplant, and cotton result from inserting a gene from the bacteria *Bacillus thuringiensis*. Gene editing is being promoted as a more precise technology that could be used to amend an existing gene rather than inserting foreign genes into an organism.

### Potential Applications

Gene editing is bringing both profoundly challenging and enabling opportunities for applications in human health, natural resource stewardship and primary production. In medicine, gene editing has already been approved for use in patients to make immune cells attack cancer cells or to mutate HIV virus DNA to stop it from replicating (Tebas et al., 2014; Reardon, 2015). In agriculture, gene editing is being used to create more hardy, nutritious and productive plants and animals (Shan et al., 2013; Wang et al., 2015). In conservation, researchers may be able to use gene editing to introduce a sterility gene into a pest as part of a pest-eradication programme, or spread a malaria resistance gene in mosquitoes (Hammond et al., 2016).

The broad range of potential applications of gene editing has the potential to re-ignite ethical debates generated by earlier forms of genetic modification. In Aotearoa New Zealand, genetically modified organisms (GMO's) have been regulated since the establishment of the Environmental Risk Management Authority (ERMA) through the Hazardous Substances and New Organisms Act (1996), responsibilities which transitioned to the Environmental Protection Authority (EPA) through the Environmental Protection Authority Act (2011). Early applications of gene technologies to create transgenic organisms were met with public outrage, leading to the establishment of a Royal Commission of Inquiry into Genetic Modification. Over 10,000 public submissions were considered by the Commission in the development of its report, which has led to no genetically modified crops being grown in New Zealand (Royal Commission on Genetic Modification, 2001). Māori were significant contributors to the debates on genetic modification and regulatory processes provide specific recognition of Māori values within decision-making processes for new organisms including GMOs (Cram et al., 2000; Environmental Protection Authority, 2016).

The variability surrounding the regulation of gene editing in the international context has led to the recent establishment of a Royal Society of New Zealand (RSNZ) Gene Editing Panel to engage the public in discussions and provide advice to the New Zealand Government on potential options for regulation (Royal Society of New Zealand, 2016, Royal Society of New Zealand, 2017a,b). Gene editing is currently considered genetic modification and therefore non-human gene-edited organisms are classified as “new organisms” and therefore are subject to approval processes under the EPA, a process which includes the incorporation of Māori perspectives.

### Literature Review

The literature suggests there are more Māori positioned on the anti-GM end of the spectrum (Gardiner, 1997; Cram et al., 2000), however a distinction is apparent between GMOs for commercial production with no clear cultural or environmental benefits and those that might provide direct community benefit (Roberts and Fairweather, 2004; Smith et al., 2013). GM projects that had a clear benefit or genuine contribution to communities and the environment were received more positively. The literature provided some consistent messages about the key Māori cultural concepts and values relevant to biotechnology and genetic research. There is a general consensus that *whakapapa* (genealogy) sits as the key concept for Māori communities. The second most commonly acknowledged cultural value is *mauri* (life essence), followed by *mana* (power/authority) and *kaitiakitanga* (guardianship). A number of other Māori terms are also used in the course of writing about Māori and biotechnology issues such as *mātauranga* (indigenous knowledge), *tikanga* (protocols), *Papatūānuku* (earth mother), and *tangata whenua* (indigenous people, literally people of the land). Culturally based concepts have also been associated with specific functions. For example, concepts related to “Consultation and Relationships” include *kawa* (customary principles), *tika* (right, correct), and *manaakitanga* (to care for, look after), while *tapu* (sacred, restricted), *taonga* (precious), *wairua* (spirit) are associated with the status of DNA and *tākoha* (gift) to the sharing of DNA (Beaton et al., 2016; Hudson et al., 2016a). Table 1 highlights the most commonly discussed Māori concepts and values.

**Table 1:** Reference to key Māori concepts.

**Concept:**	**Whakapapa**	**Mauri**	**Kaitiakitanga**	**Mana**
Te Momo, 2007	•	•	•	
Te Mata Ira (Hudson et al., 2016a)	•	•	•	•
Hutchings and Reynolds, 2005	•	•	•	
Mead, 2017	•	•		
Roberts, 2005	•	•		•
Tipene-Matua, 2006				•
Wilcox et al., 2008	•	•		•
Environmental Protection Authority, 2016		•	•	•
He Tangata Kei Tua (Hudson et al., 2016b)	•	•	•	•
Waitangi, 2011	•	•	•	

This article outlines previously articulated Māori perspectives on genetic modification, then considers the continuing influence of those cultural and ethical arguments within the context of recent developments in gene editing, and finally explore with key Māori informants how cultural values might be used to analyse the risks and benefits of gene editing in the Aotearoa New Zealand context.

## Methods

This project is a part of a New Zealand government funded research programme led by Plant & Food Research Ltd on “Turbo-breeding for New Zealand's plant industries” primarily focusing on the adoption of new breeding technologies in the horticulture sector. A component of the project explores the co-innovation interface, the interface of cultural and commercial interests and concerns, with a view to identifying processes that support Māori organizations to participate in research and commercialization activities involving gene editing technologies. The aim of this part of the project was to assess the on-going relevance of Māori concepts in the context of gene-editing. Data was collected through three key activities; a literature review; key informant interviews; and an electronic survey.

A review of 38 key publications between 2005 and 2017 provided the foundation for this project. The review covered literature relating to Māori and biotechnology, genetic modification, and genetic research with a particular focus on the Māori values, concepts and perspectives that have previously been articulated. The review became the basis for a discussion document, Māori Perspectives & Gene Editing: A Discussion Paper (Mead et al., 2017), which informed preliminary discussions with a number of agencies and Māori networks, such as the EPA's Ngā Kaihautu Tikanga Taiao (Māori Advisory Body), Te Herenga Network (National Māori Network of Iwi Environmental Practitioners), Te Tira Whakamataki (the Māori Biosecurity Network), the Biological Heritage National Science Challenge, and public consultation exercises on gene editing led by the Royal Society of New Zealand.

Ethics Approval was gained from the University of Waikato's delegated research ethics committee within the Faculty of Māori and Indigenous Studies for the collection of data from key informant interviews and an electronic survey which used the same set of open questions. The questions emerged from the key concepts identified in the literature review and focused on the potential applications and opportunities associated with gene editing, the key issues and concerns associated with gene editing, whether gene editing should be considered the same as genetic modification, and the relevance of key Māori values and concepts (Table 1) to understanding gene editing.

Eight key informants (2 × males, 6 × females) were purposefully selected from the researchers networks with interests in plant health, environmental health, human health, business, research, public understanding, and public policy to provide a range of informed Māori perspectives. Four of the key informants are active researchers albeit not in genomic sciences, and the others have strong relationships with researchers and communities. They were chosen because of the roles they play as translators between science teams and Māori communities and general familiarity with both scientific research and Māori perspectives. Key informants were interviewed separately and an electronic survey was shared with members of the Te Herenga Network (National Māori Network associated with the Environmental Protection Authority) and the SING Alumni Network (Summer Internship for Indigenous Genomics programme). The survey, which resulted in nine additional responses, was used to broaden the range of perspectives and reduce the potential bias associated with the key informant interviews. All participants were Māori and the responses to each question in the interviews (KI) and the survey (SR) were grouped and analyzed manually by the researchers using guided thematic analysis (Coffey and Atkinson, 1996). The process of coding empirical material to the research questions and emerging themes, was conducted across key domains, including potential benefits, concerns, and the relevance of Māori values and concepts.

## Results

### Interview and Survey Responses

#### What Do You See Are the Potential Applications/Opportunities Associated WITH GENE Editing?

Participants saw different opportunities for gene editing to support their communities aspirations in horticulture, conservation, maintaining the health and biodiversity of the environment, or to address human health issues. A range of potential applications were identified including preservation of endangered species of plants and animals, new health related therapies, protecting biodiversity, creating health and food security, sequencing of rare threatened and endangered endemic species (and their medical chemotypes), human health, environmental restoration, sustainable enterprise, pest control, and pest eradication. One participant noted a primary interest as ensuring that gene editing was stopped.

#### What Do You See Are the Key Issues/Concerns That Arise From Use of Gene Editing?

Participants concerns about the use of gene editing centered on the risks of adopting this technology. The risk of unintended consequences, whether it is possible to do rigorous assessments of the potential downstream effects, the reversibility of any genetic modifications, ethical considerations, and effect on kaitiaki (guardians) responsibilities were highlighted. Participants identified an innate risk from a cultural perspective that needs to be managed to limit unethical or unauthorized modifications. A number of the participants expressed a view that mixing genetic material from different species is unnatural and there was also a degree of anxiety about editing an organisms genome especially for economic gain.

An issue was raised about the benefits to society and concerns that technology favors the wealthy and tends to increase inequities through the commodification of resources. Some participants had concerns about the use of gene editing in the environment and others were more concerned about its use in humans. Specific issues were raised in relation to editing genes in the human germline because it is passed down to future generations and there is no information about the long-term effects. Risks to the environment were associated with the release of modified organisms into the environment where commercial interests exclude wider community benefits. Concerns were also expressed about the level of experimentation required before benefit emerges and how the community are kept up to date with what types of activities are underway. Fostering public conversations about genetic modification are necessary as public understanding lags well behind the current state of expert knowledge and technical capability.

In NZ law, gene editing is considered the same as genetic modification. In other countries gene editing is treated differently from GM. Do you think we have the right legal approach in NZ or do you think the law should be amended in light of the developing technology?

Strictly speaking gene-editing is a form of genetic modification and while some participants considered gene editing and genetic modification to be the same thing, others saw a spectrum of gene technologies.

“This highlights an issue regarding terminology and general understanding of genetic technologies. Genetic modification encompasses a spectrum of technologies with transgenics on one end and modern gene-editing on the other.” (Survey Response, SR7)

It was generally accepted that a regulatory regime should cover both Genetic Modification and Gene editing as a precautionary approach was necessary. The level of regulation ranged from “*No GMO in New Zealand”(SR5)* to “*Only in the laboratory and total containment” (SR6)* to “*after strict substantive and procedural decision-making*” *(SR4)*. Participants recognized the inconsistencies arising from the treatment of all genetically modified organisms in the same way and while there was some sympathy for the differences between technologies, the status quo allows all gene technologies to be monitored appropriately. Some of the participants felt that inter- and intra-specific genetic modification should be treated differently and possibly on a case by case basis. However, any change would require more consultation with Māori and the wider public to assess the effect on Māori rights and interests.

#### Do You Think Gene Editing Can Support Kaitiaki Responsibilities and Under What Circumstances?

Gene editing technologies are potentially one tool in the toolbox to protect and save species or be used to enhance health. Considerations will include the intent of the use, how kaitiaki understand the science, and whether its use disrupts or enhances the relationships they have with taonga species.

“Values-based organisations can use technologies to support their aspirations and in that respect they have a duty to explore all avenues that support kaitiaki responsibilities for taonga species.” (Key Informant; KI1)

The participants generally felt that gene editing could support kaitiaki to exercise their responsibilities and in some situations will be forced to consider extreme options like gene-editing to deal with intractable “wicked problems,” where all the choices appear on a spectrum of ethically challenging options. This might arise in terms of pest control as a tool to protect and enhance wildlife or as a way of correcting a variant of a gene known to be responsible for a disease. Where species extinctions are occurring, kaitiaki might explore gene-editing as an option. These decisions would be by hapū (sub-tribes) or iwi (tribes) as to whether this is an appropriate technology to support their responsibilities.

#### Do You Think Whakapapa Is Affected if You Introduce DNA Into One Species From Another Species? Is This the Same Case if You Edit DNA Within the Same Species?

All the participants thought that whakapapa was affected by introducing DNA from one species into another through genetic modification. Some kaumatua (elders) are against interspecies transfer while others are less concerned, and the participants expressed mixed opinions in relation to the effect of gene editing on whakapapa. Some participants felt it was dependent on the extent of the edit as some variation within the same species or sub-species is expected. The effect on whakapapa was also thought to be connected to the relationality between the species sharing or exchanging DNA. Where DNA associated with genes that are shared in different species is exchanged, this will have less impact on whakapapa. However, if a transferred gene does not have a naturally occurring sequence, then this could be seen to be cutting across whakapapa links. Some felt that gene editing definitely affects whakapapa but that could also be in a positive direction.

“Altering genes changes the genetic make-up of an individual but can be viewed similarly to an organ transplant. Whether it is ethical to change or introduce DNA into a species is another thing. For me whakapapa is lineage and your ties to whanau and your ancestors and that doesn't change with the introduction of foreign DNA. It could be viewed as enhancing your lineage to some or diluting it to others.” (SR8)

#### Is There a Difference Between Applying Gene-Editing for “Taonga Species” and Introduced or Commercially Produced Species?

There were mixed views on whether there was a difference when applying gene editing to a taonga (precious) species or an introduced or commercially produced species. It was clear that Māori should have a say in relation to both but the difference arose from the nature of the relationship Māori have with taonga species through Treaty of Waitangi obligations and indigenous rights.

“Māori should have the final say for approving editing in taonga species. For introduced or commercially produced species, all groups in NZ should be consulted (including Māori).” (SR3)

“The only difference between gene editing in taonga species and introduced or commercial species is that our responsibility to taonga species means that there is a much greater impetus to ensure minimal disruption to the whakapapa, mana, and mauri of these species.”(SR7)

Some felt that all species are interdependent and therefore taonga but would need to consider their different degrees of importance and how to deal with hybridity. Exotic species have been incorporated within rongoa Māori (traditional medicine) formulations since colonization and as such Māori walk in two worlds. Regardless of whether gene editing was being used for taonga species or other species the risks involved and ethical considerations are the same.

#### Is Mauri of a Species/Person Affected if the Gene-Edit Mimics a Natural Mutation/Variant?

The effect on mauri (life essence) represents one of the key moral dilemmas associated with genetic modification. Most people believe the life force is changed by gene editing but there were variations on this theme. Some felt that mauri was not affected if the change occurs naturally, and others mentioned that the effect on mauri can be both positive and negative. The effect on the mauri is related to the nature and size of the change including the heritability of the new characteristics. There was a concern expressed around the unintended effects of genetic variation and whether that changes the long term resilience of a species.

“Naturally occurring mutations/variants often occur due to environmental changes which enable adaptation to occur in the species. I feel a gene edit can either enhance or reduce Mauri depending on the phenotypic outcome of that species.” (SR8)

If mana is recognised through Māori leading the project and the research objectives are to benefit Māori whanau (families), hapū (sub-tribe), or iwi (tribe) are the same concerns still relevant?

There was a general belief amongst the participants that enhancing mana through Māori leadership would increase the level of engagement and acceptance to a project involving gene editing however the same concerns about gene editing still apply. There was a feeling that all gene editing, whether it be for conservation, commercial, or indigenous interests, should be subject to standardized processes even though cultural protocols are specific to each tribal authority.

“I don't believe it is necessary for Māori to lead a project, but it is critical that Māori are at least partners, to ensure that Mana is recognised.” (SR7)

Participants thought it was important to define the space and reasoning for using gene editing technology including how the project and any data/intellectual property would be managed. Māori input into this process is necessary especially for taonga species and Māori should also consider the impact of our decisions on other indigenous peoples, especially if the intended use is to eradicate a species endemic to another country.

## Discussion

A small but significant portion of research exists concerning indigenous peoples' responses to bio/nano-technology, generally establishing the 'indigenous position' as one strongly against these developments and their commercialization (Gardiner, 1997; Harry et al., 2000; Leier, 2002; Reynolds and Smith, 2002; Hutchings and Reynolds, 2005; Mead and Ratuva, 2007). The prevailing critique has been that most ‘bio/nanotechnology projects are inconsistent with Māori values, impinge on Māori rights and sovereignty, and continue a process where indigenous cultures, values, knowledge systems and even lives are marginalized and undervalued (Cram et al., 2000; Roberts and Fairweather, 2004; Cram, 2005; Hutchings and Reynolds, 2005; Te Momo, 2005; Hutchings, 2009).

Despite inclusion in existing regulatory processes and more positive interactions over the past decade (Hemara, 2006; Cheung et al., 2007; Te Momo, 2007; Hudson et al., 2012, 2016c) and the responses of participants in this project, a widespread social license for the use of gene-based technologies amongst the Māori community is unlikely in the short term. Generally, Māori do not oppose new and emerging gene editing technologies *a priori*, but instead raise concerns as to how the technologies should be used and the rationale, objectives and consequences of choosing them. Individual subjectivities inform the process as personal preferences for particular technologies are grounded in their own values, experiences and knowledge (Te Momo, 2007; Smith et al., 2016). The experience of the participants played an interesting part in the identification and management of potential risk. Sometimes those with backgrounds in particular fields, for example the environment, were more comfortable with its potential application in that domain and highlighted risks associated with other areas like health. In other cases, the reverse was true where experience in a field highlighted the specific concerns for application in that domain. The general discomfort all the participants expressed was reflected in the desire for appropriate regulation and a sense that there will always be justifiable use-cases and unpalatable use-cases. This anticipates a more dynamic approach to regulation where specific uses or types of uses are approved on a case by case basis.

Māori participation in discussions on gene technologies is as much cultural and political as scientific [(Cram, 2005), p. 62; (Hudson et al., 2010; Smith et al., 2013)]. Discussions on gene-based technologies cannot be divorced from discourse on land ownership and control over natural resources and debates traverse the spectrum of philosophical, social, ethical, and technical dimensions (Smith et al., 2013). Māori perspectives on biotechnology/genetic technologies frequently reference core cultural concepts as conceptual markers, derived from mātauranga Māori (indigenous knowledge) and tikanga Māori (Māori values), which are intrinsic to an indigenous way of viewing and living in the world. These cultural cues provide the basis for describing the cultural logic that underpins engagement in a culturally acceptable manner (Cram et al., 2000; Hudson et al., 2010, 2016c; Smith et al., 2013). This research demonstrates the cultural cues that Māori referenced in the genetic modification debate, and subsequent conversations about biotechnologies, continue to be relevant in the context of gene editing. The Māori concepts of *whakapapa* (genealogy), *mauri* (life essence), *mana* (authority), and *kaitiakitanga* (guardianship) feature prominently. *Whakapapa* and *mauri* relate to the organism itself and *mana* and *kaitiakitanga* refer to the relationship that people have with that organism. *Whakapapa* is a key reference point when talking about genetics or genomics (Hudson et al., 2016a) because it provides the foundation for how Māori construct their identities and their relationships with other species (Roberts, 2005, 2013; Hudson et al., 2007). *Mauri* relates to the distinctive and special nature of an organism including its right to life (Hudson et al., 2010; Mead, 2017). *Mana* relates to authority and provides a responsibility to act in the interests of the broader community (Mead, 2017). The expression of *kaitiakitanga* enhanced through the recognition of mana whenua status presupposes that Māori have authority over their lands and resources and that the use of gene technologies is done in ways that supports these outcomes (Thompson, 2018).

Participants in this study suggested that the effect of gene editing on Māori values is not always in a negative direction and it was suggested that whakapapa, mana, mauri, and kaitiakitanga might be enhanced through the use of gene editing technologies. This suggests that values based frameworks developed for other gene based technologies (Wilcox et al., 2008; Hudson et al., 2016c) will remain relevant in for gene editing applications. What the enhancement or diminishment of these Māori values might look like is summarized in Table 2.

**Table 2:** Impact of gene editing on Māori concepts/values.

**Value/Concept**	**Value enhancement**	**Value diminishment**
Whakapapa	Gene Editing does not involve the transfer of genes between species—**whakapapa** can be maintained and enhanced through the continued well-being of the species	Gene Editing introduces foreign DNA or involves changing the genome inter-generationally with negative consequences—**whakapapa** is diminished
Mauri	Gene Editing is being used to support human or environmental health—**mauri** is enhanced	Gene Editing is used for inappropriate purposes—**mauri** is diminished
Kaitiakitanga	Gene Editing may support or enhance resilience of ecosystems—**kaitiakitanga** is enhanced	Gene editing has unknown effects on the well-being of organisms and the ecosystem—**kaitiakitanga** is diminished
Mana	Māori are able to choose how gene editing is applied—**mana** is enhanced	Māori have no say in discussions about how gene-editing is used—**mana** is diminished
Taonga	Gene-editing supports commercial and cultural interests as identified by Māori—**Taonga** status is enhanced	Gene-Editing is used in ways that negatively affect taonga species—**Taonga** is diminished
Tapu	The use of gene editing is restricted and subject to a precautionary principle—**Tapu** is enhanced	The use of gene editing is widely approved for any purpose—**Tapu** is diminished
Wairua	Māori are involved in decision-making and are comfortable with the uses of the biotechnology—**Wairua** is enhanced	Māori are not involved in decision-making and don't know what's going on—**Wairua** is diminished
Kawa	Robust consultation and decision-making processes are developed, and Māori values inform the use of gene editing—**Kawa** is enhanced	Māori values are excluded from policy development and decision making processes—**Kawa** are diminished
Tika	Benefits of the research are shared equitably across the community—**Tika** is enhanced	Benefits are captured by commercial or special interest groups—**Tika** is diminished
Manaakitanga	Cultural protocols are developed to support the use of gene-editing—**Manaakitanga** is enhanced	No cultural support for Māori participation in gene editing activities—**Manaakitanga** is diminished
Tākoha	Recognition of Māori rights and interests to genome sequences and responsibilities associated with this—**Tākoha** is enhanced	No recognition of Māori rights, interests or responsibilities—**Tākoha** is diminished
Whanaungatanga	The use of gene editing supports a strengthening of whanau by addressing a key issue or concern—**Whanaungatanga** is enhanced	The use of gene editing does not contribute to addressing whanau issues or creates disruption in the whanau—**Whanaungatanga** is diminished

According to New Zealand law, gene editing is not deemed distinct, rather it is seen as one of many processes, tools, methods, or products of genetic modification and as such is subject to the same regulations as any other GMO. A key issue here is whether it makes sense to regulate a technology rather than regulating the outcome or product of the technology. Gene editing will allow the generation of outcomes/products similar, or identical to those generated by technologies not covered by legislation. Gene editing does not necessarily insert foreign DNA into the genome of the host organism and the DNA mutations resulting from gene editing involve small DNA sequence changes that cannot be differentiated from natural ones, even using modern sequencing technologies. The RSNZ is currently engaging the New Zealand public in debates about gene editing through a series of discussion documents (Royal Society of New Zealand, 2016; Royal Society of New Zealand, 2017a,b) and a public speaker series. The topic has recently been brought to back into the spotlight by comments from the outgoing NZ Chief Government Science Advisor who suggested that while the use of gene technologies continued to be heavily debated, from a scientific point of view “*There are no significant ecological or health concerns associated with the use of advanced genetic technologies*,” and that we need to engage society in debate that is “*more constructive and less polarized than in the past*.” (Science Media Centre, 2018) (https://www.sciencemediacentre.co.nz/2018/07/02/changing-of-the-chief-scientist-guard-in-the-news/). The participants in this study wanted to engage in a constructive discussion to create a robust regulatory framework that addresses gene editing on a case by case basis and utilizes Māori values within the decision-making process.

## Summary

Gene editing is the most recent gene based technology promising benefits across health, environmental, and commercial domains. It emerges in the wake of decades old, ethically charged, debates about GMOs and transgenic applications which seared controversy about gene technologies into the public consciousness. As the New Zealand government considers whether to change the regulations around gene editing technologies it is supporting a new round of public consultation exercises. While Māori have expressed strong views and opposition to genetically modified organisms in the past, it is important to assess the continuing influence of those perspectives.

The participants demonstrated that Māori values and cultural concepts continue to inform Māori perspectives on biotechnology and their regulation. Whakapapa, mauri, mana, and kaitiakitanga provide a cultural scaffold for considering the philosophical, moral, ethical and technical dimensions relevant to the use of gene editing technologies. It is apparent that a range of views exist across the Māori community and that participants are prepared to consider the use of gene editing on a case dependent basis, especially where it aligns with Māori worldviews. Incorporating Māori values into decision-making processes could provide a balancing factor to ensure broader community interests remain a key consideration in the future use of gene editing technologies. The application of gene editing technologies heightens societal sensitivities about inequities as their use tends to prioritize commercial interests over community benefit (Smith, 2016). However, further research is required to characterize the strength of the various positions identified in this pilot study and to explore its relevance to other indigenous communities.

## Author Contributions

MH: contribution to framing and writing, analysis of surveys and interviews, primary editor. AM: primary data collector, contribution to framing and writing, analysis of literature, and development of tables. DC: contribution to framing and writing of scientific context, review of analysis and manuscript. NR, SM, and PW: data collection, review of analysis and manuscript. AA: contribution to writing of scientific context, review of analysis and manuscript, principal investigator.

### Conflict of Interest Statement

DC and AA are employed by The New Zealand Institute for Plant and Food Research. The remaining authors declare that the research was conducted in the absence of any commercial or financial relationships that could be construed as a potential conflict of interest.

## Figures and Tables

**Table:** 

*Aotearoa*	New Zealand
*Hapū*	Sub-tribal grouping
*Hui*	Tribal meetings
*Iwi*	Tribe
*Kaitiakitanga*	Guardianship
*Kawa*	Customary principles
*Mana*	Power/authority
*Manaakitanga*	To care for/to look after
*Mātauranga*	Indigenous knowledge
*Mauri*	Life essence
*Ngā Kaihautu Tikanga Taiao* Māori advisory body for Environmental Protection Authority	
*Papatūānuku*	Earth mother
*Rongoa Maori*	Traditional medicine
*Tākoha*	Gift
*Tangata whenua*	People of the land
*Taonga*	Precious
*Tapu*	Sacred/restricted
*Te Ao Māori*	Māori worldview
*Te Herenga Network*	National Māori Network of Environmental Practitioners
*Te Tira Whakamātaki*	Māori Biosecurity Network
*Tika*	Right/correct
*Tikanga*	Protocols
*Wānanga*	Traditional learning spaces and activities
*Whakapapa*	Genealogy
*Whānau*	Family, includes extended family
*Wairua*	Spirit
